# Supplementary material for: Widespread adaptive evolution during repeated evolutionary radiations in New World lupins
Source: Nat Commun. 2016 Aug 8;7:12384. doi: 10.1038/ncomms12384 (PMC4979066; doi:10.1038/ncomms12384)
Supplement: Supplementary Information — Supplementary Figures 1 - 10, Supplementary Tables 1 and 2, Supplementary Notes 1 and 2, Supplementary Methods and Supplementary References [file ncomms12384-s1.pdf]

## Supplementary Figures

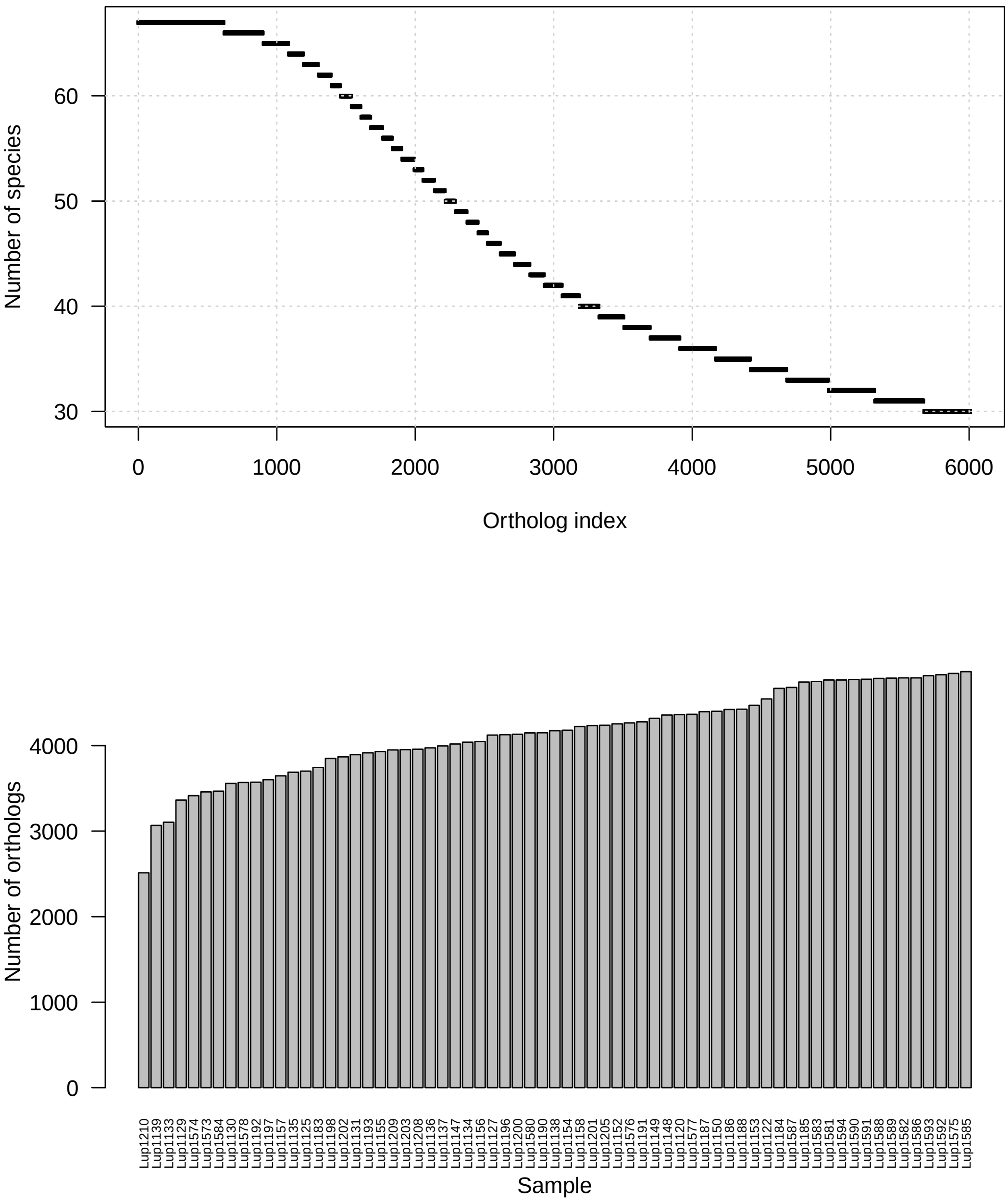


**Supplementary Figure 1**

Summary results of orthology inference. Top panel shows the number of species sampled for each orthologous gene, bottom panel the number of orthologous genes recovered for each lupin sample (sample accession names as in Supplementary Data 1).

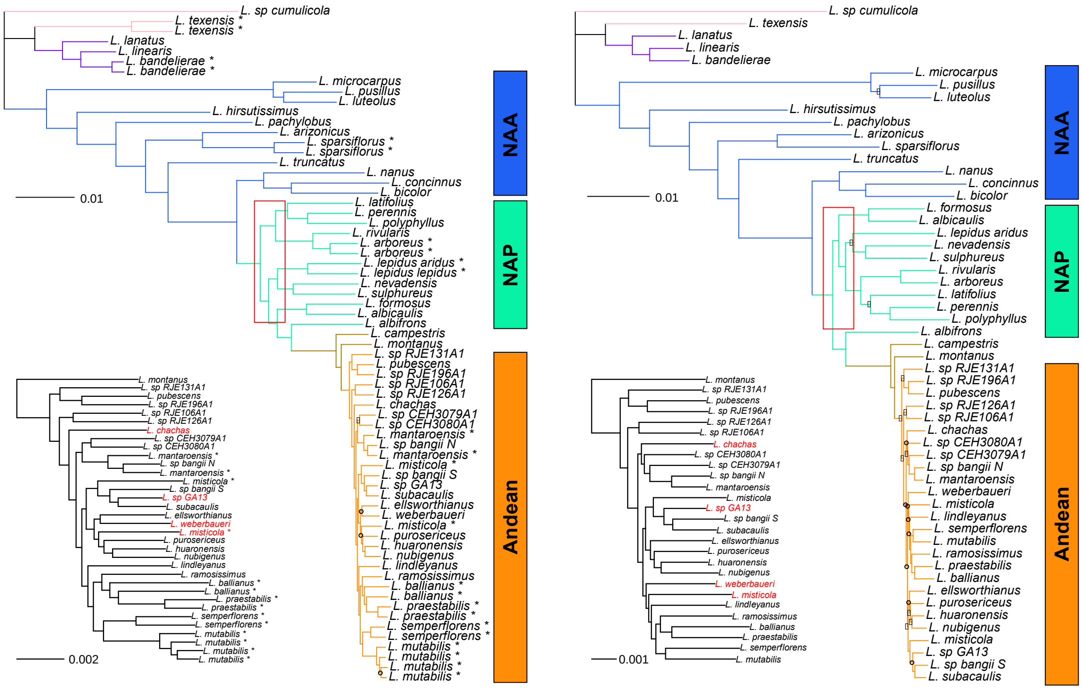


## Supplementary Figure 2

Comparison of ML-based (*left*) and coalescent-based phylogenies (*right*), based on 6,013 orthologous genes. Red boxes indicate the differing placements of the North American perennial species. For each phylogeny, the bottom-left insets show detailed phylogenetic relationships within the Andean clade, with different placements of species between the supermatrix and species-tree reconstructions highlighted in red. In the ML phylogeny species represented by more than one accession are marked with asterisks. Nodes with bootstrap support above 90 but below 100 are marked with filled circles; those with bootstrap support below 90 are marked with empty circles; all other nodes have bootstrap support of 100. The *Lupinus* lineages compared in this work are highlighted: North American Annual lineages (NAA), North American Perennial lineages (NAP) and Andean lupins.

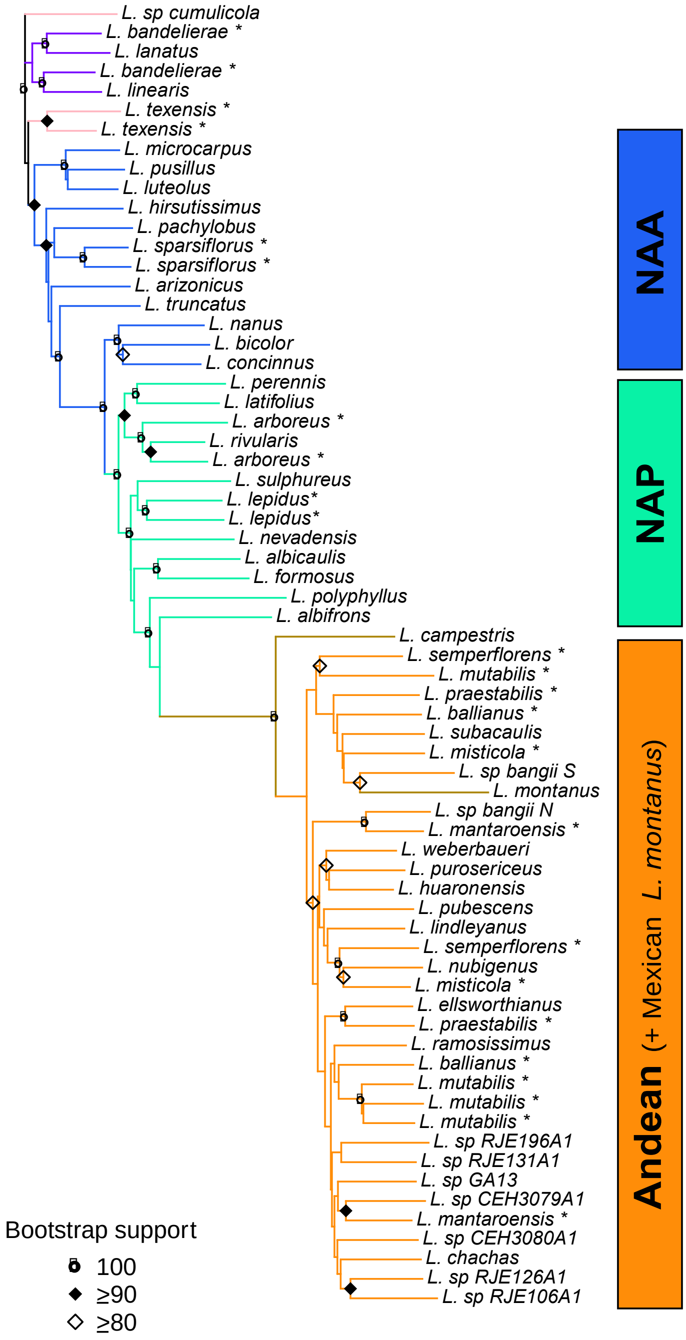


**Supplementary Figure 3**

Neighbour-joining tree obtained using pairwise gene expression distances between samples, calculated as $1 - \rho$ where $\rho$ is Spearman's correlation coefficient between normalized expression levels of 6,013 genes. Bootstrap values obtained with 100 replicates. Species represented by more than one accession are marked with asterisks. Main lineages compared in this work are highlighted: North American Annual lineages (NAA), North American Perennial lineages (NAP) and Andean lupins.

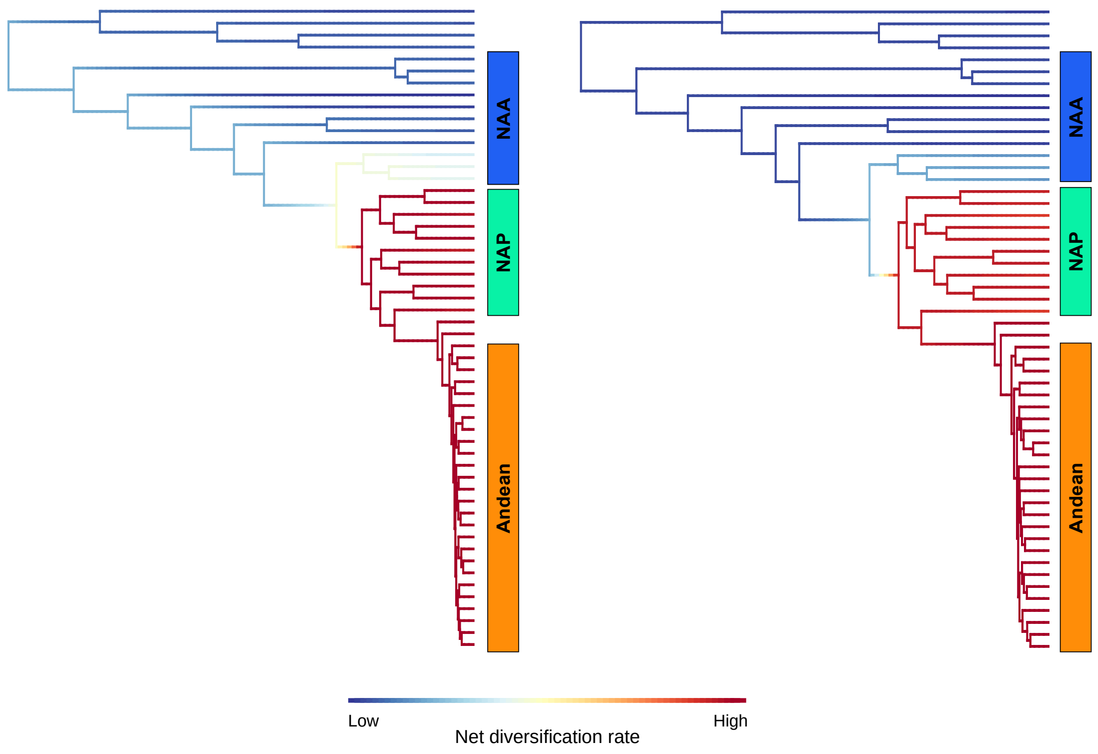


## Supplementary Figure 4

Net diversification rates estimated for each branch using the ML (*left*) or coalescent-based (*right*) phylogenies. Colours depict estimated net diversification rates per branch. The *Lupinus* lineages compared in this work are highlighted: North American Annual lineages (NAA), North American Perennial lineages (NAP) and Andean lupins.

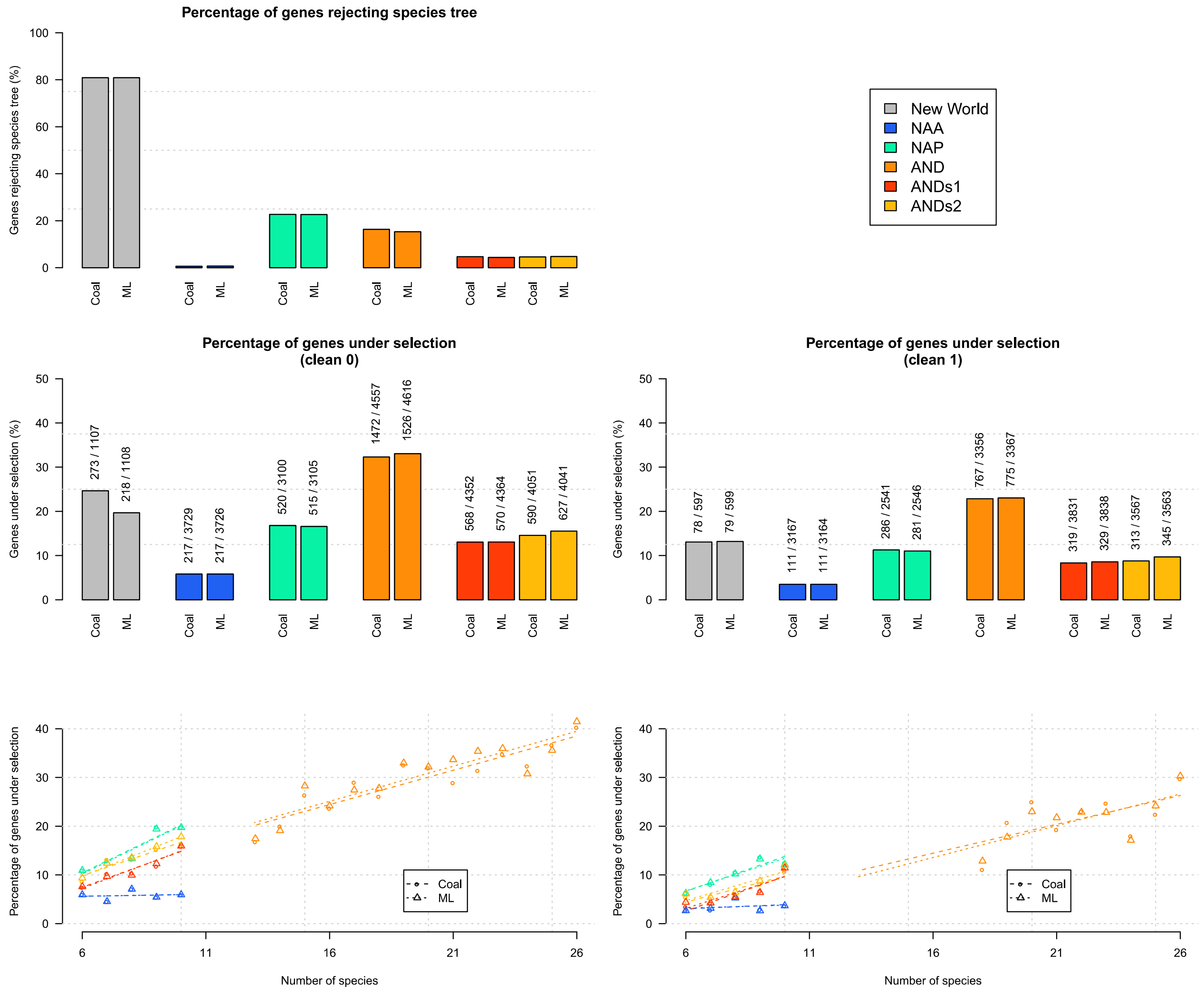
Percentage of genes rejecting species tree
Genes rejecting species tree (%)
0
20
40
60
80
100
Coal
ML
New World
NAA
NAP
AND
ANDs1
ANDs2
Percentage of genes under selection (clean 0)
Genes under selection (%)
0
10
20
30
40
50
273 / 1107
218 / 1108
217 / 3729
217 / 3726
520 / 3100
515 / 3105
1472 / 4557
1526 / 4616
568 / 4352
570 / 4364
590 / 4051
627 / 4041
Percentage of genes under selection (clean 1)
78 / 597
79 / 599
111 / 3167
111 / 3164
286 / 2541
281 / 2546
767 / 3356
775 / 3367
319 / 3831
329 / 3838
313 / 3567
345 / 3563
Percentage of genes under selection
Number of species
6
11
16
21
26
Coal
ML

**Supplementary Figure 5**

Results of the analysis of selection on coding sequences using sites-models in PAML, with different topologies: Maximum-likelihood (ML) and coalescent-based trees (Coal). *Top left* – Percentage of genes excluded from dN/dS tests due to phylogenetic incongruence (SH-test, FDR correct $P < 0.05$). *Middle* – Percentage of genes tested that preferred a model including sites evolving under positive selection, over the simpler model where all sites evolve neutrally or under purifying selection (FDR corrected $P < 0.05$ in comparison of Models M8 vs M7 and M8 vs M8a in paml). Numbers above bars denote numbers of genes under selection / tested. *Bottom* – The effect of the number of species tested on the percentage of genes exhibiting some sites evolving under positive selection. For *middle* and *bottom* panels, left graphs show results obtained when using ambiguous codons after removing species and codons with more than 80% missing data (cleandata=0 in paml), right graphs the results when using only fully known codons (cleandata=1 in paml). Groups tested: New World – all species sampled; NAA – North American Annual lineages; NAP – North American Perennial lineages; AND – Andean species; ANDs1 and ANDs2 – subsets of 10 randomly selected Andean species.

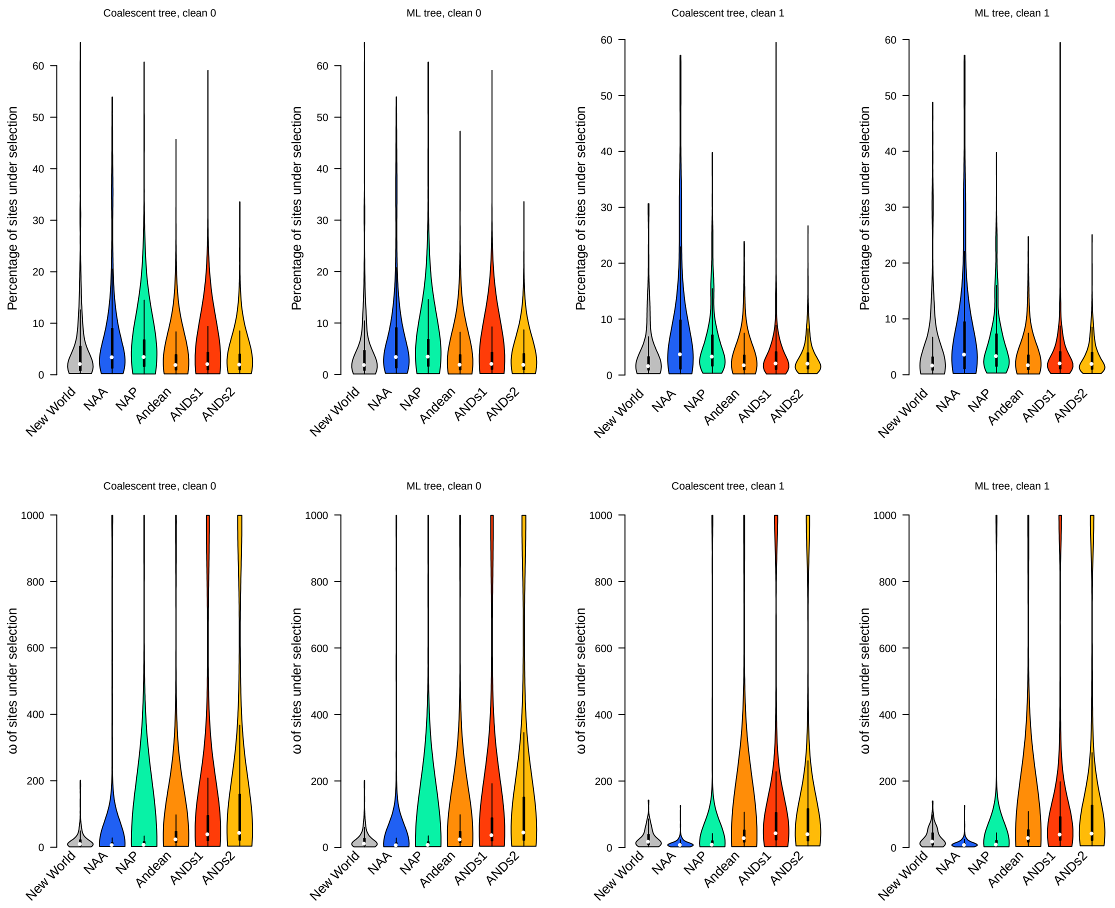


## Supplementary Figure 6

The percentage of sites under selection (top) and dN/dS ratios for these sites (bottom), across all genes showing evidence of positive selection. We show the results when using the ML (columns 2 and 4) or coalescent based (columns 1 and 3) topologies, and different missing data stringency (as in Supplementary Fig. 5).

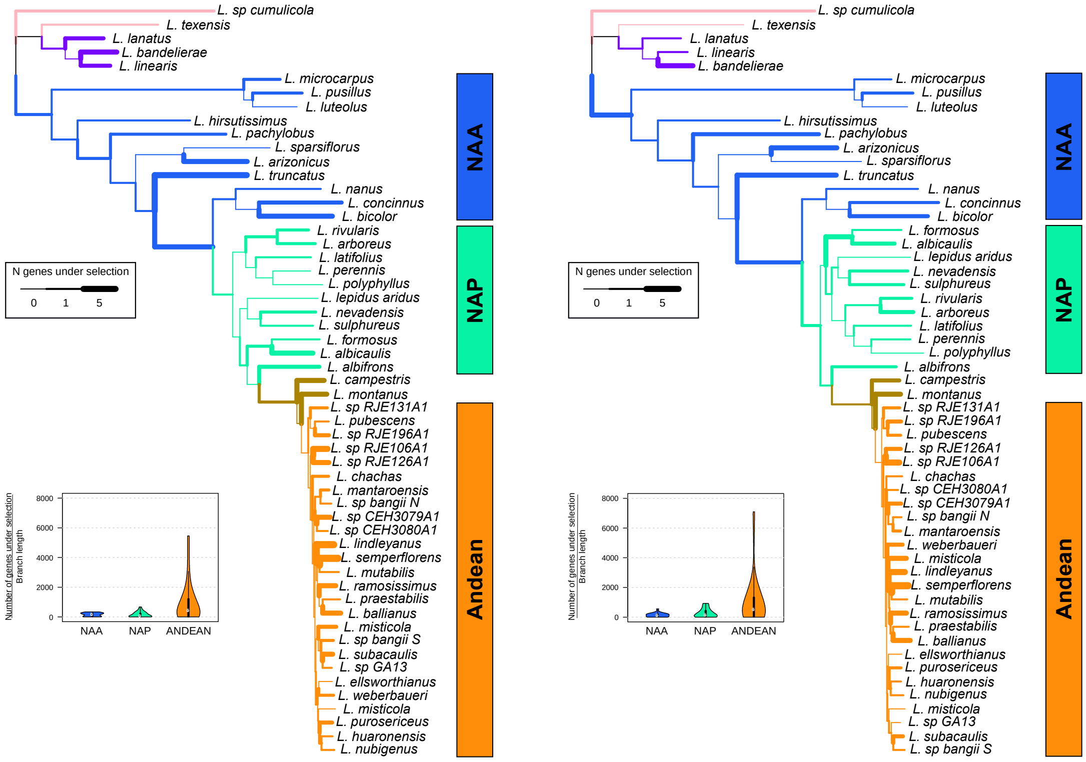


**Supplementary Figure 7**

Results of the analysis of selection on coding sequences using branch-site models in HYPHY, with different topologies: Maximum-likelihood (*left*) and coalescent-based (*right*). Width of branches denotes the number of genes inferred to have experienced episodic positive selection. Inset graphs depict the distribution of the number of genes under selection per branch for the main lineages studied (NAA – North American Annual lineages; NAP – North American Perennial lineages; AND – Andean clade) after normalisation for branch lengths.

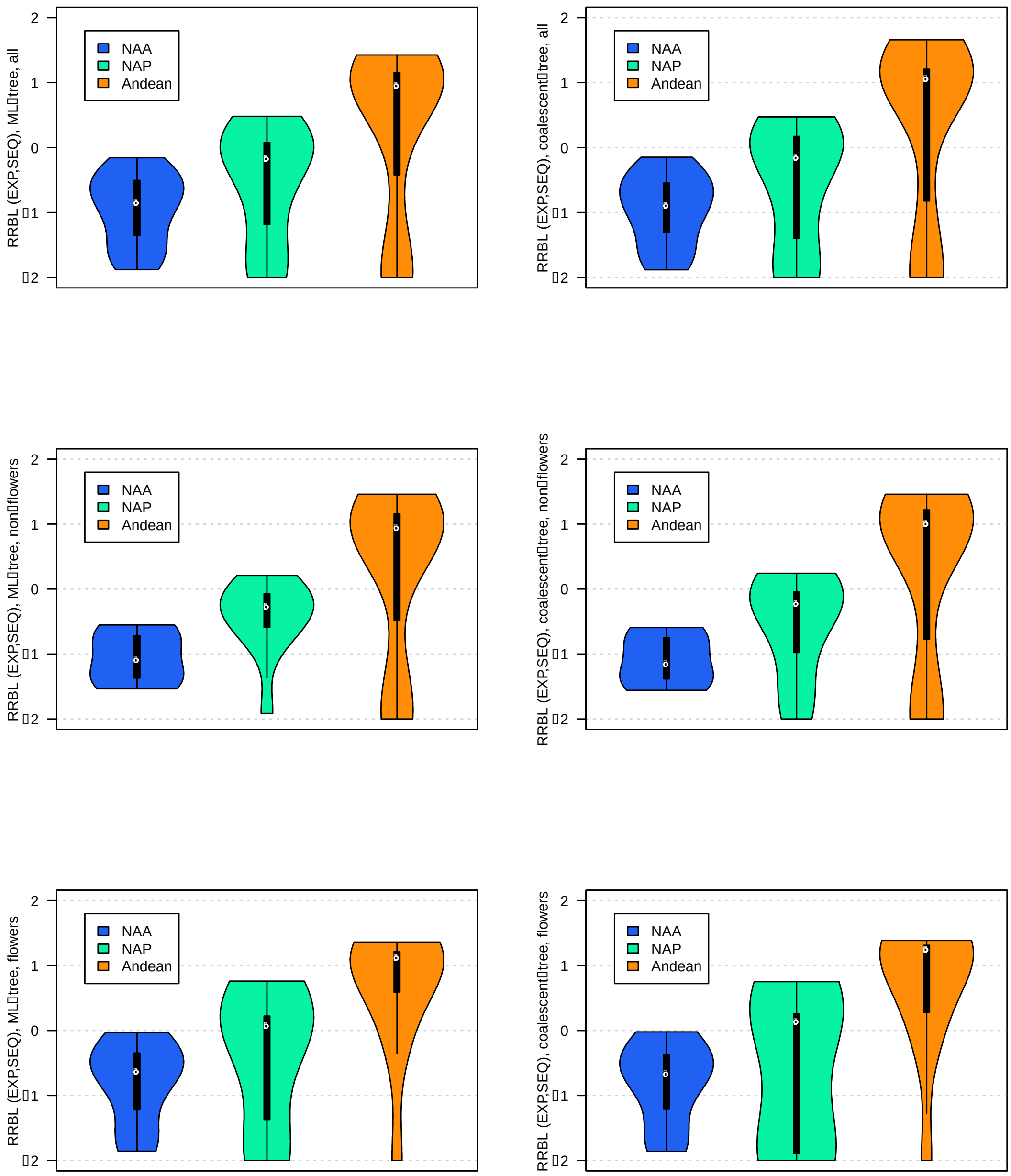


**Supplementary Figure 8**

Distribution of rescaled relative branch lengths (RRBL) per branch when using the Maximum-Likelihood (*left*) or coalescent-based (*right*) topologies, and all samples (*top*), only samples without flower tissue (*middle*) or only samples with flower tissue (*bottom*).

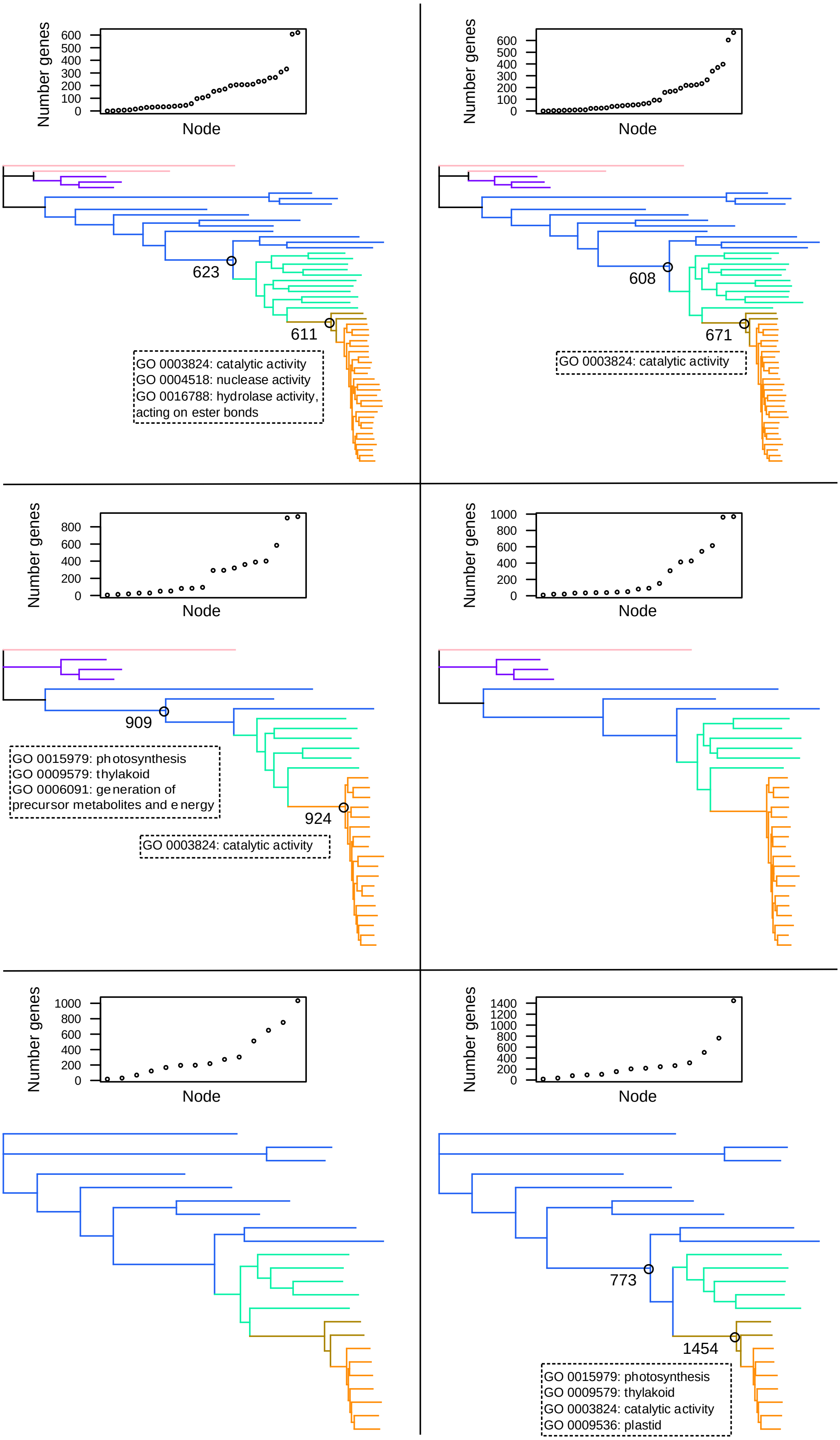

Number genes
Node
623
611
GO 0003824: catalytic activity
GO 0004518: nuclease activity
GO 0016788: hydrolase activity, acting on ester bonds
608
671
GO 0003824: catalytic activity
909
GO 0015979: photosynthesis
GO 0009579: thylakoid
GO 0006091: generation of precursor metabolites and energy
924
GO 0003824: catalytic activity
773
1454
GO 0015979: photosynthesis
GO 0009579: thylakoid
GO 0003824: catalytic activity
GO 0009536: plastid

**Supplementary Figure 9**

Results of the analysis of shifts in optimal gene expression values of individual genes using OU-models, with different phylogenies: Maximum-Likelihood (*left*) and coalescent-based (*right*). Top row shows results using all species sampled, middle row species for which only stem and leave tissue was available, and bottom row results for species with stem, leave and flower tissues available. For each panel, top graph depicts the number of genes showing a shift in optimal gene expression values on different nodes; and bottom phylogeny highlights nodes with unusually high number of genes showing a shift in optimal gene expression value (more than 1.5 times the inter-quartile range above the third quartile of the distribution). Gene Ontology terms over represented (Fisher's exact test, FDR corrected $P < 0.05$) in the set of genes showing shifts in gene expression are depicted next to each outlier node (not shown when no over represented GO terms were found).

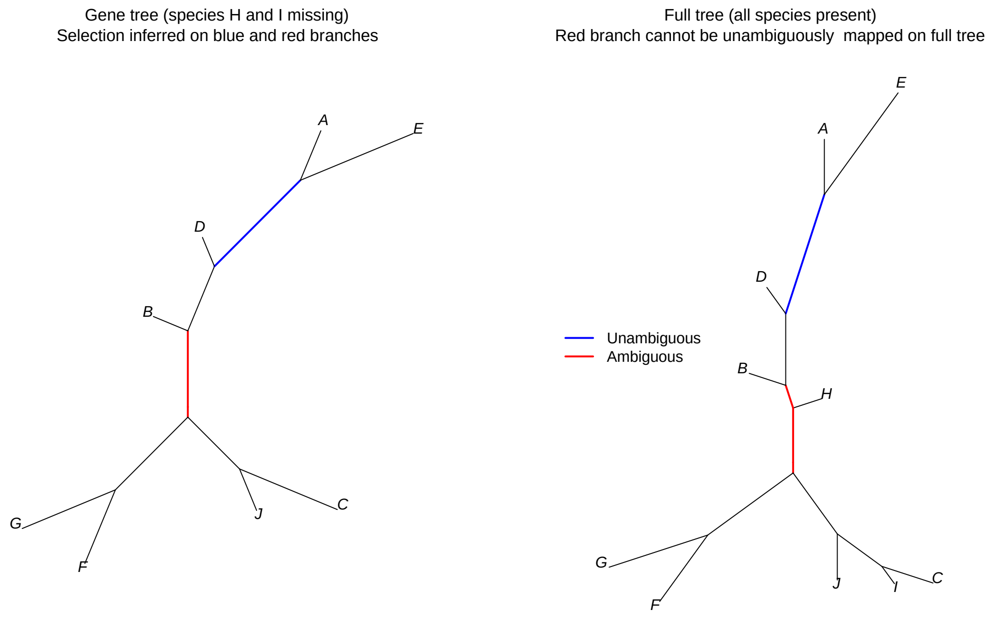


**Supplementary Figure 10**

Example result of the analysis of branch-site models in HYPHY. Due to missing data, different genes were sampled on a variable number of species, and it was sometimes impossible to unambiguously assign specific gene-branch combinations unto the full species tree. The left tree represents an example result where selection on a gene was inferred in two branches (red and blue). Because species *H* was not sampled in this gene, the red branch is ambiguously mapped unto the full species tree (*right*). Results mentioned in the main text refer only to branches that could be unambiguously mapped unto the species tree.

## Supplementary Tables

## Supplementary Table 1

Phylogenetic analysis of selection on coding sequences in *Lupinus* spp.

| Group tested | SH-test (%) * | Genes tested # | Genes under selection (N) $ | Genes under selection (%) $ |
|---|---|---|---|---|
| **New World** | 80.9% | 1,107 | 273 | 24.7% |
| **NAA** | 0.6% | 3,729 | 217 | 5.8% |
| **NAP** | 22.7% | 3,100 | 520 | 16.8% |
| **Andean** | 16.3% | 4,557 | 1,472 | 32.3% |
| **ANDs1** | 4.7% | 4,352 | 568 | 13.1% |
| **ANDs2** | 4.5% | 4,051 | 590 | 14.6% |

(*****) Percentage of genes rejecting the species tree in favour of the transcript tree (SH-test FDR-corrected $P < 0.05$). (#) Number of genes passing all filters (see methods) and tested for the presence of sites evolving under positive selection. ($) Number (and percentage) of genes tested which preferred a model allowing for some sites evolving under positive selection to a model allowing only for purifying selection and neutrality (FDR-corrected $P < 0.05$).

**Supplementary Table 2**

Phylogenetic analysis of selection on coding sequences in different plant genera.

| Genus | N ‡ | SH-test (%) * | Genes tested # | Genes under selection (N) $ | Genes under selection (%) $ |
|---|---|---|---|---|---|
| ***Lupinus* § (NAA)** | 8 | 7.2% | 10,828 | 369 | 3.4% |
| ***Lupinus* § (NAP)** | 8 | 42% | 8,759 | 1,376 | 15.7% |
| ***Lupinus* § (Andean)** | 8 | 16.6% | 7,717 | 800 | 10.4% |
| ***Flaveria*** | 8 | 19% | 6,679 | 104 | 1.6% |
| ***Glycine*** | 6 | 23.8% | 1,344 | 58 | 4.3% |
| ***Helianthus*** | 7 | 15.9% | 7,588 | 399 | 5.3% |
| ***Linum*** | 8 | 1.9% | 5,263 | 37 | 0.7% |
| ***Oryza*** | 8 | 22.1% | 5,999 | 277 | 4.6% |
| ***Populus*** | 6 | 12.2% | 276 | 7 | 2.5% |
| ***Solanum*** | 8 | 1.8% | 6,129 | 29 | 0.5% |

(‡) Number of species included in analysis. (*) Percentage of genes rejecting the species tree in favour of the transcript tree (SH-test FDR-corrected $P < 0.05$). (#) Number of genes passing all filters (see methods) and tested for the presence of sites evolving under positive selection. ($) Number (and percentage) of genes tested which preferred a model allowing for some sites evolving under positive selection to a model allowing only for purifying selection and neutrality (FDR-corrected $P < 0.05$). (§) Results shown are for random subsets of 8 species from each main Lupinus subgroup analysed (NAA – North American Annuals; NAP – North American Perennials; and Andean clade).

## Supplementary Notes

### Supplementary Note 1

The ML phylogenetic reconstruction based on the analysis of all 6,013 genes returned a robustly supported topology (only 4 branches with bootstrap support below 100, Supplementary Fig. 2). Of the 11 species with multiple accessions available, eight were resolved as monophyletic and two as closely related but paraphyletic. Conversely, two accessions of the Andean *L. misticola* were placed in different Andean subclades, despite overall similarity in morphology and relatively close geographical proximity of these two accessions from southern Peru. Given the lack of a comprehensive taxonomic account for Andean lupins, we treat the two accessions of *L. misticola* as belonging to different (cryptic) species pending further evidence.

Phylogenetic reconstruction using coalescent-based methods returned a similar topology to the supermatrix approach, albeit with lower bootstrap support values (Figure 1 and Supplementary Fig. 2). The differences between the coalescent and ML based topologies were (i) differing placements of four Andean species; and (ii) the phylogenetic relationships of the North American Perennial lupins, which formed a paraphyletic grade comprising two (in the coalescent-based tree) or four (in the ML tree) lineages.

Given the large amount of data analysed, the incongruence between the two sequence-based phylogenetic approaches (supermatrix and species-tree) is likely to stem from incomplete lineage sorting (ILS) or gene flow between species [1,2]. The very high diversification rates in New World lupins [3,4] suggest ILS is likely to be prevalent causing conflicting phylogenetic signal among gene trees within this group. As the coalescent-based phylogenetic approach directly deals with ILS [5], we favour the topology obtained with this approach over the ML method. In the main text we discuss only results using the coalescent-based topology, but we performed all following analyses using either of the two phylogenies independently. Results were very similar throughout, and are presented in the Supplementary Figures.

Phylogenetic reconstruction using gene expression values resulted in a very similar overall topology to the sequence-based approaches, although one of the two Mexican species was resolved within the Andean clade and only 3 of the 11 species with multiple accessions were resolved as monophyletic (Supplementary Fig. 3). The close match between the overall topologies obtained with sequence data and

expression values confirms that changes in gene expression values can be used to infer phylogenetic relationships. However, the lack of support for relationships between species within each clade suggests that accumulation of neutral divergence in gene expression values occurs over longer evolutionary time, and cannot be used to infer relationships in very recent clades.

**Supplementary Note 2**

Using the branch-site models implemented in HYPHY, we identified 204 instances of episodic positive selection affecting specific branches and genes (164 tips and 40 internal branches), of which 146 cases could be unambiguously mapped to the species tree (Supplementary Fig. 7). Only three branches exhibited positive selection on five or more genes, and all were terminal tips in the fast radiating Andean lineage. Of the 146 unambiguously mapped cases, 97 were found in the rapidly diversifying Andean and NAP lineages (Pearson's Chi-squared test, NAP+Andean versus remaining tree, $P = 0.87$). However, the number of genes under selection within different lineages was not significantly different. This likely reflects the strong dependence of the branch-site tests on branch length, with short branches lacking the evidence needed to detect even frequent or strong selection [6]. When normalised by branch length the rapidly diversifying Andean clade showed much higher rates of positive selection compared to NAA, while NAP exhibited intermediate values (Supplementary Fig. 7, inset graph). Results using the ML topology were very similar (Supplementary Fig. 7).

## Supplementary Methods

**Orthology inference.** To obtain alignments of orthologous genes across all species we used a clustering and phylogenetic-based orthology inference method [7]. Python scripts used to automate the following steps were obtained from https://bitbucket.org/yangya/phylogenomic_dataset_construction.

In a first step, we reduced the redundancy of each individual transcriptome by clustering the CDSs using CD-HIT-EST v 4.6 [8] with a sequence identity threshold of 0.99. We then performed an all-by-all blast with BLASTN v 2.2 [9] using the CDSs of all individuals as both query and subject. We used all default values with BLASTN and

retained up to 1000 best hits for each sequence. We trimmed sequences to remove ends that did not have any hit to any other sequence. To remove hits to conserved motifs and short sequence fragments we excluded blast hits where the length of the alignment was smaller than 1/3 of the length of either the query or the subject sequences. Blast hits were clustered with MCL [10] with an inflation value of 1.4. Clusters with less than 30 tips were discarded, and the remaining clusters were aligned with MAFFT v 7.123b [11] using the accuracy-oriented method E-INS-i and a maximum of 1000 iterative refinement cycles.

We performed two rounds of refinement on the resulting multiple-species sequence alignments to remove assembly and clustering errors [7]. Each round consisted of the following steps: sequence alignments were trimmed with PHYUTILITY [12] to remove positions with more than 10% missing data; cleaned alignments were used in RAXML v8.0.1 [13] to estimate a phylogenetic tree with the GTR + GAMMA model of nucleotide substitution; tips longer than 10 times the average of its sisters, or longer than 0.7 expected substitutions per site, were removed; mono- and paraphyletic tips belonging to the same individual were removed, keeping only the tip with the most unambiguous characters; internal branches longer than 1 expected substitution per site (0.75 for the second round) were cut, and only subtrees with 30 or more species kept.

We then pruned the homologous gene trees to obtain orthologous genes using the Maximum Inclusion method [7]. For each homologous gene tree, we searched for the subtree with the maximum number of non-repeating taxa, and with at least 30 tips. This subtree was extracted and kept as an orthologous gene tree, and the search repeated with the remainder of the homologous tree until no subtree with at least 30 non-repeating tips could be found. Tips in the resulting orthologous gene trees were trimmed as before to exclude misassembled sequences (tips were cut if longer than 10 times its sisters or longer than 0.7 expected substitutions per site). Finally, for each orthologous gene we extracted the aligned sequences from the homologous genes and re-aligned them with PRANK v.140110 [14] using the codon substitution matrix method [15]. Using this method we identified 6,013 orthologous genes, which were present in at least 30 accessions, with 66% of all accessions sampled in over 66% of genes (Supplementary Fig. 1).

**Phylogenetic inference.** For the Maximum-Likelihood (ML) phylogenetic reconstruction, each gene was treated as a separate partition and we used RAXML to perform 100 rapid bootstrap replicates [16] followed by a thorough ML search. We used the General Time Reversible nucleotide substitution model (GTR, [17]) with rate heterogeneity between sites modelled with the CAT approximation (GTR+CAT, [18]).

For the coalescent-based phylogenetic reconstruction, we followed the statistical binning method to cluster the orthologous genes into "supergenes" [19]. We used RAXML with the GTR substitution model and gamma distributed rate heterogeneity (GTR+Γ, [20]) to obtain bootstrap support for individual gene trees. We used a bootstrap support of 75 as a threshold to evaluate conflict between gene trees and built an incompatibility graph, from which genes were combined into bins using a balanced heuristic method [19] (software available from http://www.cs.utexas.edu/users/phylo/datasets/binning/). We concatenated the sequence alignments of each gene in the same bin into "supergenes", and obtained "supertrees" for each of these concatenated datasets with RAXML (GTR+Γ model, 100 bootstrap replicates). We then used ASTRAL v 4.7.7 [21] to estimate the species tree that agrees with the largest number of quartets induced by the set of most likely "supertrees", and the bootstrap "supertrees" to infer support values for this tree (100 bootstraps). For species with more than one individual sampled multiple accessions were coded as belonging to the same species during tree search with ASTRAL. The coalescent-based tree returned by ASTRAL does not include branch lengths, which are required for some of the analyses performed subsequently. To obtain branch lengths for this tree we optimised branch lengths and parameters of the GRT+CAT model (but fixed the topology) in RAXML using the same settings as with the "supermatrix" approach.

To obtain an expression-based phylogeny we used the neighbour-joining method on a matrix of pairwise gene expression values between samples (for details see section '*Estimation of gene expression levels*'). We estimated the support for the inferred phylogeny with 100 bootstraps (resampling genes and re-estimating distance matrix for each bootstrap replicate).

**Estimation of gene expression levels.** To obtain comparable across-species gene expression values for each gene, we used BOWTIE2 v 2.2 [22] to map the trimmed raw reads of each individual to its own *de novo* reference transcriptome, and RSEM v 1.2 [23]

to estimate the relative gene expression level of each gene (expressed in transcripts per million, TPM, [24]). We log2-transformed the TPM values and used the Poisson model of [25] as implemented in the R package POISSONSEQ v 1.1 to normalize the relative expression values across samples. For species with multiple conspecific individuals sampled we used the average TPM values across accessions.

**Comparison with other plant genera.** To understand how the amount of selection detected within *Lupinus* compares to other plant genera we analysed previously published transcriptome data, obtained from the Short Read Archive (http://www.ncbi.nlm.nih.gov/sra). We analysed genera for which data from at least six species were available: *Flaveria*, *Glycine*, *Helianthus*, *Linum*, *Oryza*, *Populus* and *Solanum* (Supplementary Data 2). We analysed a maximum of eight and minimum of six species per genus.

Illumina raw reads were downloaded and processed as described for the newly collected data from *Lupinus*: trimming, transcriptome assembly, orthology detection, phylogenetic inference, phylogenetic conflict and analysis of dN/dS "sites models". To account for differences in power due to sample size, we randomly selected and reanalysed eight species from each of the *Lupinus* lineages analysed (NAA, NAP and Andean clade). For each orthologous gene of each genus we compared the fit of the models M8 and M7 with a Likelihood Ratio Test (LRT) with 2 degrees of freedom, and corrected resulting p-values with the FDR method. Our results show that selection is two to three times more common in the fast diversifying *Lupinus* lineages (NAP and Andean clade) compared to any other genus analysed (main text, Figure 2C and Supplementary Table 2).

## Supplementary References


1 Pamilo, P. & Nei, M. Relationships between gene trees and species trees. *Mol Biol Evol* **5**, 568-583 (1988).

2 Maddison, W. P. Gene Trees in Species Trees. *Syst Biol* **46**, 523-536 (1997).

3 Drummond, C. S., Eastwood, R. J., Miotto, S. T. S. & Hughes, C. E. Multiple continental radiations and correlates of diversification in Lupinus (Leguminosae): Testing for key innovation with incomplete taxon sampling. *Syst Biol* **61**, 443-460 (2012).

4 Hughes, C. & Eastwood, R. Island radiation on a continental scale: exceptional rates of plant diversification after uplift of the Andes. *Proc Natl Acad Sci USA* **103**, 10334-10339 (2006).

5 Degnan, J. H. & Rosenberg, N. A. Gene tree discordance, phylogenetic inference and the multispecies coalescent. *Trends Ecol Evol* **24**, 332-340 (2009).

6 Smith, M. D. *et al.* Less Is More: An Adaptive Branch-Site Random Effects Model for Efficient Detection of Episodic Diversifying Selection. *Mol Biol Evol* **32**, 1342-1353 (2015).

7 Yang, Y. & Smith, S. A. Orthology Inference in non-model organisms using transcriptomes and low-coverage genomes: Improving accuracy and matrix occupancy for phylogenomics. *Mol Biol Evol* **31**, 3081-3092 (2014).

8 Li, W. & Godzik, A. Cd-hit: a fast program for clustering and comparing large sets of protein or nucleotide sequences. *Bioinformatics* **22**, 1658-1659 (2006).

9 Camacho, C. *et al.* BLAST+: architecture and applications. *BMC Bioinformatics* **10**, 421 (2009).

10 van Dongen, S. *Graph Clustering by Flow Simulation* Ph.D thesis thesis, University of Utrecht, (2000).

11 Katoh, K. & Standley, D. M. MAFFT multiple sequence alignment software version 7: improvements in performance and usability. *Mol Biol Evol* **30**, 772-780 (2013).

12 Smith, S. A. & Dunn, C. W. Phyutility: a phyloinformatics tool for trees, alignments and molecular data. *Bioinformatics* **24**, 715-716 (2008).

13 Stamatakis, A. RAxML version 8: a tool for phylogenetic analysis and post-analysis of large phylogenies. *Bioinformatics* **30**, 1312-1313 (2014).

14 Loytynoja, A. & Goldman, N. An algorithm for progressive multiple alignment of sequences with insertions. *Proc Natl Acad Sci USA* **102**, 10557-10562 (2005).

15 Kosiol, C., Holmes, I. & Goldman, N. An empirical codon model for protein sequence evolution. *Mol Biol Evol* **24**, 1464-1479 (2007).

16 Stamatakis, A., Hoover, P. & Rougemont, J. A rapid bootstrap algorithm for the RAxML Web servers. *Syst Biol* **57**, 758-771 (2008).

17 Tavaré, S. in *American Mathematical Society: Lectures on Mathematics in the Life Sciences* Vol. 17 57-86 (Amer Mathematical Society, 1986).

18 Stamatakis, A. in *Parallel and Distributed Processing Symposium, 2006. IPDPS 2006. 20th International.* 8 pp.

19 Mirarab, S., Bayzid, M. S., Boussau, B. & Warnow, T. Statistical binning enables an accurate coalescent-based estimation of the avian tree. *Science* **346**, 1250463 (2014).

20 Yang, Z. Maximum likelihood phylogenetic estimation from DNA sequences with variable rates over sites: approximate methods. *J Mol Evol* **39**, 306-314 (1994).

21 Mirarab, S. *et al.* ASTRAL: genome-scale coalescent-based species tree estimation. *Bioinformatics* **30**, i541-548 (2014).

22 Langmead, B. & Salzberg, S. L. Fast gapped-read alignment with Bowtie 2. *Nat Methods* **9**, 357-359 (2012).

23 Li, B. & Dewey, C. N. RSEM: accurate transcript quantification from RNA-Seq data with or without a reference genome. *BMC Bioinformatics* **12**, 323 (2011).

24 Wagner, G., Kin, K. & Lynch, V. Measurement of mRNA abundance using RNA-seq data: RPKM measure is inconsistent among samples. *Theory Biosci* **131**, 281-285 (2012).

25 Li, J., Witten, D. M., Johnstone, I. M. & Tibshirani, R. Normalization, testing, and false discovery rate estimation for RNA-sequencing data. *Biostatistics* **13**, 523-538 (2012).
